# Urinary metabolomics reveals potential biomarkers for monitoring carbon black exposure-related airway injury

**DOI:** 10.3389/fpubh.2026.1809269

**Published:** 2026-06-25

**Authors:** Rou Wen, Zijing Wu, Zhaohui Mu, Jianzhong Zhang, Yaozu Han, Yixuan Wang, Jinglong Tang, Yuxin Zheng, Wei Han, Weiwei Qin

**Affiliations:** 1 School of Medicine and Pharmacy, Ocean University of China, Qingdao, China; 2 Qingdao Key Laboratory of Respiratory Comorbidity Remodeling and Precision Prevention, Qingdao Hospital, University of Health and Rehabilitation Sciences (Qingdao Municipal Hospital), Qingdao, China; 3 Department of Respiratory and Critical Care Medicine, Qingdao Hospital, University of Health and Rehabilitation Sciences (Qingdao Municipal Hospital), Qingdao, China; 4 Central Laboratory, Qingdao Hospital, University of Health and Rehabilitation Sciences (Qingdao Municipal Hospital), Qingdao, China; 5 Department of Environmental and Occupational Health, School of Public Health, Qingdao University, Qingdao, China; 6 Department of Anesthesiology, Qingdao Hospital, University of Health and Rehabilitation Sciences (Qingdao Municipal Hospital), Qingdao, China

**Keywords:** biomarkers, carbon black, metabolomics, occupational exposure, small airway injury

## Abstract

**Background:**

Carbon black (CB) exposure is a well-established cause of pulmonary injury, yet sensitive and practical biomarkers for early detection remain lacking. This study aims to address this gap. Here, we investigate whether urinary metabolomics can provide noninvasive signatures for the early identification and risk stratification of CB–associated airway injury.

**Methods:**

In 2018, we enrolled 45 CB–exposed packing workers from a CB factory in Henan Province and 45 municipal waterworks employees without occupational particulate exposure as controls from the same city. After accounting for environmental confounding, participants completed baseline questionnaires; internal exposure dose, lung function, and airway structure were assessed, and urine was collected concurrently. Urinary metabolomes were quantified by UPLC-Orbitrap-MS, and covariate-adjusted linear regression identified metabolites associated with CB exposure and airway remodeling, which informed development of an exposure-related small-airway injury prediction model.

**Results:**

CB-exposed workers showed significantly higher lung CB burden, impaired pulmonary ventilatory function (reduced FEF_25_ and FEF_25_P), and increased 6th-generation airway wall thickness (WA%), with untargeted metabolomics identifying 210 differential metabolites primarily enriched in the purine metabolism pathway. Correlation analysis between differential metabolites and 6th-generation WA% (LB1+2) further identified four key endogenous metabolites, namely creatine, citric acid, Prostaglandin E2, and 3 beta-Hydroxy-5-cholestenoate. A KNN classifier incorporating these four metabolites demonstrated mean AUCs of 0.782 ± 0.06 and 0.631 ± 0.138 in the training and test sets, respectively.

**Conclusions:**

Urinary metabolomics identified four candidate biomarkers for carbon black-related airway injury, and a metabolite-based predictive model may offer a noninvasive, cost-effective approach for early screening in occupationally exposed populations.

## 1 Introduction

Carbon black (CB, CAS No. 1333-86-4) is an elemental carbon particulate substance formed by the incomplete combustion or thermal decomposition of hydrocarbons (1). As a classic artificially synthesized nanomaterial, it possesses superior performances including reinforcement, wear resistance, high-temperature resistance, anti-aging property, electrical conductivity and colorability (2). Accordingly, CB has been extensively used in tires and other rubber products, plastics, batteries, printing inks and coatings (3–5). Fueled by rising global market demand, the worldwide output of CB hit 13.59 million tons in 2022. Among this, China's production volume reached 5.948 million tons, occupying 43.8% of the global total and ranking first across the world. With the sustained expansion of production scale, occupational exposure risks have markedly increased for employees engaged in CB manufacturing and downstream industrial processing (6).

Prior studies in CB-exposed workers have established that CB primarily enters the human body via the respiratory tract, whereupon inhaled particles deposit and accumulate within pulmonary tissue. Their inherent resistance to metabolic clearance facilitates prolonged biopersistence, ultimately contributing to the development of a spectrum of respiratory pathologies (7). Acute inhalation exposure has been shown to trigger pulmonary inflammatory responses, while chronic occupational exposure may precipitate more significant respiratory impairment, including pulmonary fibrosis (8, 9). Prolonged CB exposure has furthermore been significantly associated with decrements in spirometric indices such as forced expiratory volume in 1 s (FEV_1_) and forced vital capacity (FVC) (10), and has been closely linked to small airway wall thickening (11)—a cardinal manifestation of small airway disease, which is itself recognized as an important precursor lesion in the pathogenesis of chronic obstructive pulmonary disease (COPD) (12). Critically, early-stage small airway injury is frequently subclinical, rendering timely detection particularly challenging. Current clinical approaches for monitoring small airway injury rely predominantly on pulmonary function testing and high-resolution computed tomography (HRCT); however, spirometric results are susceptible to inter-individual variability in patient cooperation and operator technique (13), while HRCT entails cumulative ionizing radiation exposure (14), collectively limiting their suitability for repeated screening and longitudinal surveillance in occupational settings. Accordingly, there is a pressing need for simple, noninvasive, and repeatable monitoring strategies, together with validated specific biomarkers, to enable the early detection and systematic surveillance of CB exposure-associated small airway injury.

Metabolomics is a well-established systems-biology approach for comprehensive profiling of endogenous metabolites and for identifying disease- and exposure-related biomarkers (15). It offers high sensitivity, real-time capture of dynamic biochemical responses, and streamlined sample processing, making it suitable for large-scale population screening. Urinary metabolomics, in particular, is noninvasive and enables repeated sampling. It has been widely applied to biomarker discovery in COPD (16), asthma (17), and other respiratory diseases, and has been shown to reflect metabolic responses to air pollutants such as carbon monoxide (CO) and particulate matter (PM) (18). Nevertheless, urinary metabolomic biomarkers specifically linked to CB exposure remain insufficiently studied. Even rarer are multi-metabolite signatures and predictive models that can be translated into actionable tools for risk stratification. These gaps motivate a framework that integrates urinary metabolomic signals into an interpretable, testable risk-prediction system.

Here, we integrate urinary metabolomics with quantitative airway phenotypes and internal dose metrics to derive and validate a parsimonious biomarker signature and a risk-prediction model for CB-related small-airway injury. Leveraging an established occupational cohort with CB exposure in Henan Province (19), untargeted urinary metabolomics was employed to characterize CB exposure-associated metabolic perturbations, identify endogenous metabolites correlated with quantitative indices of airway structure, and develop a predictive model for CB-related small airway injury. This framework enables scalable and noninvasive risk stratification and early screening among exposed populations, providing a scientific basis for the surveillance and prevention of particulate matter-related respiratory damage.

## 2 Material and methods

### 2.1 Study population and exposure assessment

This study was nested within an occupational cross-sectional survey initiated in 2018 at a CB factory and a waterworks facility in the same city in Henan Province, China. The exposure group comprised 45 packing workers with direct CB contact and ≥1 year of continuous employment. Control group comprised 45 waterworks employees without occupational particulate exposure. Participants in both groups resided in comparable areas, thereby reducing environmental confounding. All participants provided written informed consent. The study protocol was approved by the Ethics Committee of the National Institute of Occupational Health and Poison Control, Chinese Center for Disease Control and Prevention (Approval No. NIOHP-201604).

The exposure assessment methodology has been described in detail previously (20). Physicochemical characterization by scanning electron microscopy and transmission electron microscopy revealed that the CB used in this study (purity > 99.8%) comprises spherical primary particles of 30–50 nm in diameter, which further self-assemble into larger aggregates and agglomerates spanning hundreds of nanometers, without evidence of intermediate hybrid morphologies. Systematic and repeated environmental monitoring was conducted in carbon black packing (CBP) workshops and corresponding reference areas, encompassing airborne PM_2.5_ mass concentrations, PM_2.5_-bound elemental carbon (EC), organic carbon (OC), total carbon (TC), and particle size distribution. In 2018, the geometric mean PM_2.5_ concentration in CBP workshops reached 637.4 μg/m^3^, with an associated EC concentration of 364.6 μg/m^3^ (21). The EC/TC ratio consistently exceeded 92.5% across all sampling campaigns, affirming that the particulate matter in these occupational settings is compositionally dominated by elemental carbon—a defining characteristic of CB aerosols.

### 2.2 Internal dose assessment

Carbon content in airway macrophages (CCAM) measured from induced sputum was used as a target-site internal dose biomarker of CB exposure (11). Briefly, participants inhaled 4.5% sterile hypertonic saline via an ultrasonic nebulizer (8 L/min) in three 5-min sessions separated by 3-min intervals and expectorated sputum, which was collected into sterile tubes, fixed with Saccomano's solution, and stored cool and protected from light. Sputum samples were first centrifuged at 4 °C to obtain cell pellets, followed by enzymatic liquefaction using the commercial sputum digestive reagent Sputasol™ (Thermo Fisher Scientific) at a 1:1 volume ratio relative to the original sputum. The liquefied samples were subsequently filtered and re-centrifuged to isolate airway cells for downstream analysis. The cell suspension was resuspended in DPBS, prepared as smear slides, air-dried, and stained with Diff-Quik. Under 1,000 × oil-immersion light microscopy, 50 well-preserved macrophages per participant were randomly imaged; ImageJ was used to quantify the proportion of cytoplasmic area occupied by carbon particles for each cell, and the upper quartile of PCOC across the 50 cells was defined as CCAM for that participant. Image acquisition and analysis were performed blinded to ensure objectivity and reproducibility (Figure S1).

### 2.3 Pulmonary function test

Spirometry was performed in accordance with ATS/ERS standards using a calibrated CHESTGRAPH HI-101 spirometer without bronchodilator administration. Each participant's height, weight, and basic demographic information were recorded prior to testing. Participants rested for 10 min in an upright seated position before performing at least three technically acceptable FVC maneuvers, with the highest reproducible values retained for analysis. Data were saved and the procedure was repeated for subsequent participants. Recorded indices included FVC, FEV_1_, maximal mid-expiratory flow (MMEF), and forced expiratory flows at 25%, 50%, and 75% of FVC (FEF_25_, FEF_50_, and FEF_75_).

### 2.4 High-resolution computed tomography

Whole-lung HRCT scans were acquired in the supine position using a GE 64-slice spiral CT scanner (120 kVp; 0.6 s/rotation; automatic tube current; pitch 0.985; slice thickness 0.625 mm). Images were stored in DICOM format and processed using an airway analysis algorithm in Philips ISP software. Four segmental bronchi — LB1+2, LB9, RB1, and RB9 — were selected for quantitative airway analysis. Measurements were obtained at two standardized cross-sectional locations: the orifice of the 6th-generation bronchus and the midpoint extending to the subsequent generation. The key structural parameter derived from these measurements was the 6th-generation airway wall area percentage (WA%), serving as a quantitative index of airway wall remodeling.

### 2.5 Urinary metabolomics and data processing

Prior to metabolomic profiling, a portion of each urine sample was retained for creatinine quantification by enzymatic colorimetry using an automatic biochemical analyzer. All participants had normal renal function. The remaining urine samples were pretreated using a 96-well high-throughput protein precipitation method. Briefly, thawed samples were vortexed, randomized, and mixed with pre-chilled LC–MS grade acetonitrile at a volume ratio of 1:3. The mixtures were vortexed for 1 min, incubated at −20 °C for 30 min, and centrifuged at 12,000 rpm for 15 min at 4 °C. The resulting supernatants were vacuum-dried below 30 °C, reconstituted in 100 μl of 2% aqueous acetonitrile, and recentrifuged at 10,000 rpm for 10 min at 4 °C before injection.

Instrument calibration was conducted before analysis using a standard mixture (Thermo Fisher) to ensure mass accuracy within ±5 ppm. A pooled quality control (QC) sample was prepared by mixing equal volumes of all study samples and was injected every 10 injections to monitor analytical repeatability. The relative standard deviation (RSD) of retention time was consistently below 1%. The coefficient of variation (CV) of metabolite peak areas was calculated from QC samples; metabolites with CV > 30% were excluded from further analysis to ensure data quality.

Chromatographic separation was performed on an Omic High resolution Ultra sprayer column (75 μm × 15 cm, C18) maintained at 60 °C. Mobile phase A was water containing 0.1 % formic acid, and mobile phase B was 80 % acetonitrile containing 0.1 % formic acid. The flow rate was programmed as follows: 0–14 min, 1 μl/min; 14–14.2 min, 3 μl/min; 14.2–14.4 min, 3 μl/min; 14.4–14.6 min, 3 μl/min; 14.6–14.9 min, 3 μl/min; 14.9–15 min, 2 μl/min (washing step). The gradient elution profile was: 0–14 min, 5%−25 % B; 14–14.2 min, 25%−90 % B; 14.2–14.4 min, hold at 90 % B; 14.4–14.6 min, 90%−2 % B; 14.6–14.9 min, hold at 2 % B. The injection volume was 2 μl.

Mass spectrometry was performed using an Orbitrap Exploris™ 480 mass spectrometer (Thermo Fisher Scientific) operated in positive ion mode. Full-scan spectra were acquired over the range m/z 350–1,200 at a resolution of 120,000 for full scan and 30,000 for data-independent acquisition (DIA). Higher-energy collisional dissociation (HCD) was set to a normalized collision energy of 32 %, with an automatic gain control target of 1 × 10^6^ and a maximum injection time of 50 ms. The radio frequency lens (RF lens) was set to 50 %. The source temperature was 350 °C, the ion transfer tube temperature was 320 °C, sheath gas flow was 40 arbitrary units, auxiliary gas flow was 10 arbitrary units, and the spray voltage was 3.2 kV.

Raw metabolomic data were first processed in R software and subsequently subjected to normalization and missing value imputation in MetaboAnalyst 6.0. All metabolite abundances were finally normalized to urinary creatinine to correct for urine dilution variation. Variables with a missing value ratio greater than 50% across all samples were excluded, and the remaining missing values were imputed via the k-nearest neighbor (KNN) imputation algorithm, followed by log_2_ transformation to enhance feature comparability. All annotated metabolites were sorted by Description in ascending order and by Score and Fragmentation score in descending order, and duplicates with identical descriptions were removed. Finally, metabolite annotations were screened with cut-off thresholds of Score > 40 and Fragmentation score > 50 for quality control.

### 2.6 Statistical analysis

Questionnaire and clinical data were entered into EpiData 3.0. Linear regression was used to evaluate the independent associations of CB exposure with pulmonary function and airway structural indices, as well as for between-group comparisons, adjusting for age, BMI, smoking pack-years, and alcohol drinking status where appropriate. Differential metabolites were defined using *P* < 0.05 and fold change > 1.5 or fold change < 0.67. PCA and OPLS-DA were used for pattern recognition, with 200-permutation testing to assess model robustness. Kyoto Encyclopedia of Genes and Genomes (KEGG) pathway enrichment analysis was performed to infer biological functions. Linear regression analyses were performed to assess two categories of associations: the correlation between metabolites and CB exposure, and the association between airway morphological parameters and differentially expressed endogenous metabolites. Metabolites demonstrating significant associations with both CB exposure and airway remodeling were designated as core metabolites and incorporated into subsequent predictive model development.

To assess model stability and generalizability, a repeated random-splitting validation strategy was employed. The entire dataset was randomly partitioned into training and test sets at a 7:3 ratio across 100 independent iterations, using stratified sampling to preserve the class distribution. In each iteration, a KNN model was developed exclusively on the training set, after standardizing all features. The number of neighbors (k) was selected via 5-fold cross-validation within the training set, optimizing the area under the receiver operating characteristic curve (AUC). Potential confounders — including age, BMI, smoking pack-years, and alcohol drinking status — were incorporated as covariates together with the four metabolites. The held-out test set was reserved solely for independent performance evaluation, without any involvement in model training or parameter selection. Model discrimination was quantified by AUC, computed separately for the training and test sets at each iteration. The mean and standard deviation of AUC values across all 100 iterations were subsequently reported to characterize model performance and its variability. To provide a comprehensive visualization of overall predictive performance, aggregated prediction probabilities pooled across all iterations were further used to construct composite ROC curves for both the training and test sets. All statistical analyses were performed in R (version 4.4.2).

## 3 Results

### 3.1 Participant characteristics

A total of 90 participants were enrolled, including 45 in the CB-exposed group and 45 in the control group. Baseline characteristics collected in 2018 showed no statistically significant differences between the two groups in age, BMI, smoking status, or pack-years of smoking (Table 1). A significant difference was observed in alcohol drinking status (*P* = 0.030), which was therefore adjusted for in subsequent analyses. Overall, the major potential confounders were comparable between the groups. Meanwhile, the CCAM level, a marker of internal CB burden, was significantly higher in the CB-exposed group (median, 10.57%) than in the control group (median, 1.38%; *P* < 0.001), indicating a markedly higher internal CB burden in exposed workers.

**Table 1:** General characteristics and CB exposure status of the study subjects.

Variables	Control group (*n* = 45)	CB-exposed group (*n* = 45)	*p*-value
Demographics	
Age (yr, Mdn, Q1, Q3)	47 (42, 50)	47 (45, 48)	0.453^c^
BMI (kg/m^2^, M ± SD)	26.25 ± 3.80	25.28 ± 3.32	0.200^a^
Smoking status	
Never smoked (*n*, %)	9, 20.0	6, 13.3	0.514^b^
Smoking (*n*, %)	29, 64.4	34, 75.6	
Quit smoking (*n*, %)	7, 15.6	5, 11.1	
Pack-years of smoking (Mdn, Q1, Q3)	19.37(5.5, 24)	8.25(1.65, 16.5)	0.061^c^
Alcohol drinking status	
Non-drinking (*n*, %)	8, 17.8	1, 2.2	0.030^d^
Drinking (*n*, %)	37, 82.2	44, 97.8	
CCAM (%, Mdn, Q1, Q3)	1.38 (1.19, 2.25)	10.57 (7.49, 17.28)	< 0.001^c^

^a^Student's t-test; ^b^Chi-square test; ^c^Mann-Whitney U-test; ^d^Fisher's exact test.

### 3.2 CB-induced pulmonary function impairment

To evaluate the association between CB exposure and pulmonary function, spirometry was used to compare lung function indices between the control and CB-exposed groups (Table 2). Compared with the control group, the CB-exposed group showed downward trends in conventional spirometric measures (FVC, FEV_1_) and multiple airway-related indices including MMEF, FEF_50_, and FEF_75_; however, most of these differences did not reach statistical significance (all *P* > 0.05), except for MMEF (*P* = 0.050) and PEF *(P* = 0.047). Notably, forced expiratory flow at 25% of forced vital capacity (FEF_25_) was significantly decreased in the CB-exposed group (β = −0.85, 95%CI: −1.46–−0.23, *P* = 0.007), and its percent-predicted value (FEF_25_P) was also significantly reduced (β = −9.97, 95%CI: −17.69–−2.25, *P* = 0.012). These findings suggest that CB exposure primarily affects airway ventilatory function, with particularly pronounced impairment in early-phase expiratory flow, while conventional lung function indices (FVC, FEV_1_) did not show significant decrements.

**Table 2:** Effects of CB exposure on airway ventilation function.

Variables	Control group	CB-exposed group	β	95% CI	*p*-value
FVC (L, M ± SD)	3.80 ± 0.49	3.74 ± 0.60	−0.08	[−0.31, 0.16]	0.514
FEV_1_ (L, M ± SD)	3.31 ± 0.40	3.24 ± 0.53	−0.13	[−0.32, 0.07]	0.199
MMEF (L/s, M ± SD)	4.13± 1.00	3.84 ± 0.99	−0.45	[−0.89, 0.00]	0.050
MMEFP (%, M ± SD)	96.9 ± 23.1	89.3 ± 22.4	−9.30	[−19.77, 1.16]	0.081
PEF (L/s, M ± SD)	7.54 ± 1.42	7.02 ± 1.30	−0.64	[−1.26, −0.01]	0.047
PEFP (%, M ± SD)	85.5 ± 16.2	80.1 ± 14.0	−6.13	[−13.16, 0.89]	0.086
FEF_25_ (L/s, M ± SD)	7.09 ± 1.41	6.34 ± 1.27	−0.85	[−1.46, −0.23]	0.007
FEF_25_P (%, M ± SD)	89.8 ± 17.8	80.6 ± 15.6	−9.97	[−17.69, −2.25]	0.012
FEF_50_ (L/s, M ± SD)	4.90 ± 1.32	4.55 ± 1.27	−0.52	[−1.10, 0.07]	0.083
FEF_50_P (%, M ± SD)	90.8 ± 23.7	84.6 ± 23.9	−8.28	[−19.08, 2.52]	0.131
FEF_75_ (L/s, M ± SD)	2.06 ± 0.78	1.93 ± 0.74	−0.28	[−0.60, 0.05]	0.094
FEF_75_P (%, M ± SD)	83.2 ± 31.9	75.9 ± 24.8	−10.13	[−23.23, 2.97]	0.128

Multiple linear regression models were all adjusted for age, BMI, pack-years of smoking, and alcohol drinking status.

### 3.3 CB-induced airway remodeling

HRCT-based quantification of 6th-generation bronchi revealed structural alterations in the CB-exposed group compared with controls (Table 3). A significant increase in airway wall area percentage (WA%) was observed specifically in the LB1+2 segment (β = 6.89, 95%CI: 1.85–11.93, *P* = 0.008), while other segments (LB9, RB1, RB9) showed no statistically significant differences (all *P* > 0.05). Collectively, these findings indicate that CB exposure is associated with increases in WA%, particularly in the LB1+2 segment, supporting the deleterious effects of occupational CB exposure on airway structural integrity.

**Table 3:** Effects of CB exposure on WA% of the 6th-generation airways.

Variables	Control group	CB-exposed group	β	95% CI	*p*-value
6th WA%	
LB9 (%, M ± SD)	53.56 ± 11.44	55.07 ± 14.64	3.71	[−2.33, 9.74]	0.225
RB1 (%, M ± SD)	54.30 ± 13.10	54.08 ± 13.27	2.96	[−3.03, 8.95]	0.328
LB1 + 2 (%, M ± SD)	55.43 ± 12.46	59.56 ± 10.12	6.89	[1.85, 11.93]	0.008
RB9 (%, M ± SD)	54.51 ± 12.27	59.07 ± 12.31	5.22	[−0.54, 10.97]	0.075

Multiple linear regression models were all adjusted for age, BMI, pack-years of smoking, and alcohol drinking status.

### 3.4 Characterization of urinary metabolomic profiles among CB exposed workers

**Figure 1:**
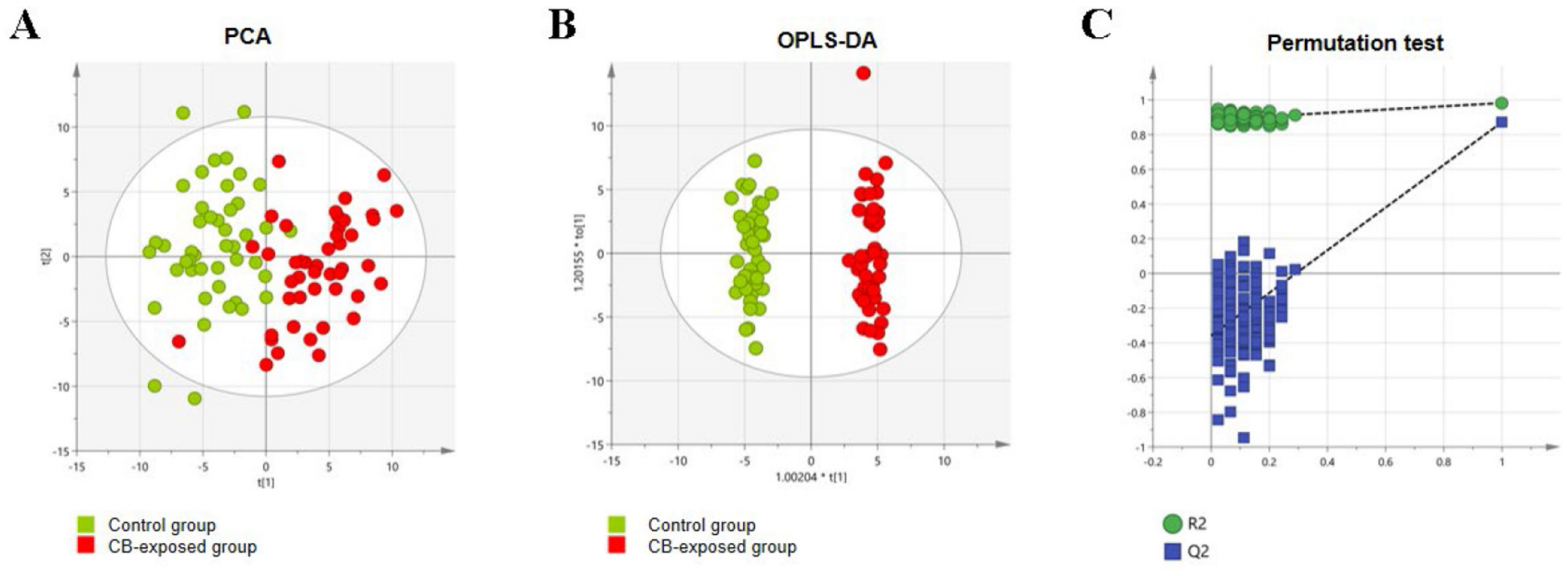
Multivariate statistical analyses of urinary metabolomic profiles in the CB-exposed and control groups.**(A)** PCA score plot depicting global variation in urinary metabolic profiles. **(B)** OPLS-DA score plot illustrating group separation between CB-exposed and control participants. **(C)** Permutation testing for OPLS-DA model validation, demonstrating model robustness and excluding overfitting.

A total of 1,101 metabolites were annotated by UPLC–MS. Unsupervised PCA showed an apparent separation trend in metabolic profiles between the CB-exposed and control groups, with most samples falling within the 95% confidence ellipse (Figure 1A), consistent with systematic between-group differences. After excluding metabolites with >50% missingness, 751 robust features were retained for downstream analyses. The OPLS-DA further discriminated the groups (Figure 1B). Validated by 200 permutation tests yielded a negative *Q*^2^ intercept (Figure 1C), indicating no overfitting, and demonstrating the reliability of the discriminant ability.

### 3.5 Alterations in urinary metabolic profiles associated with CB exposure

**Figure 2:**
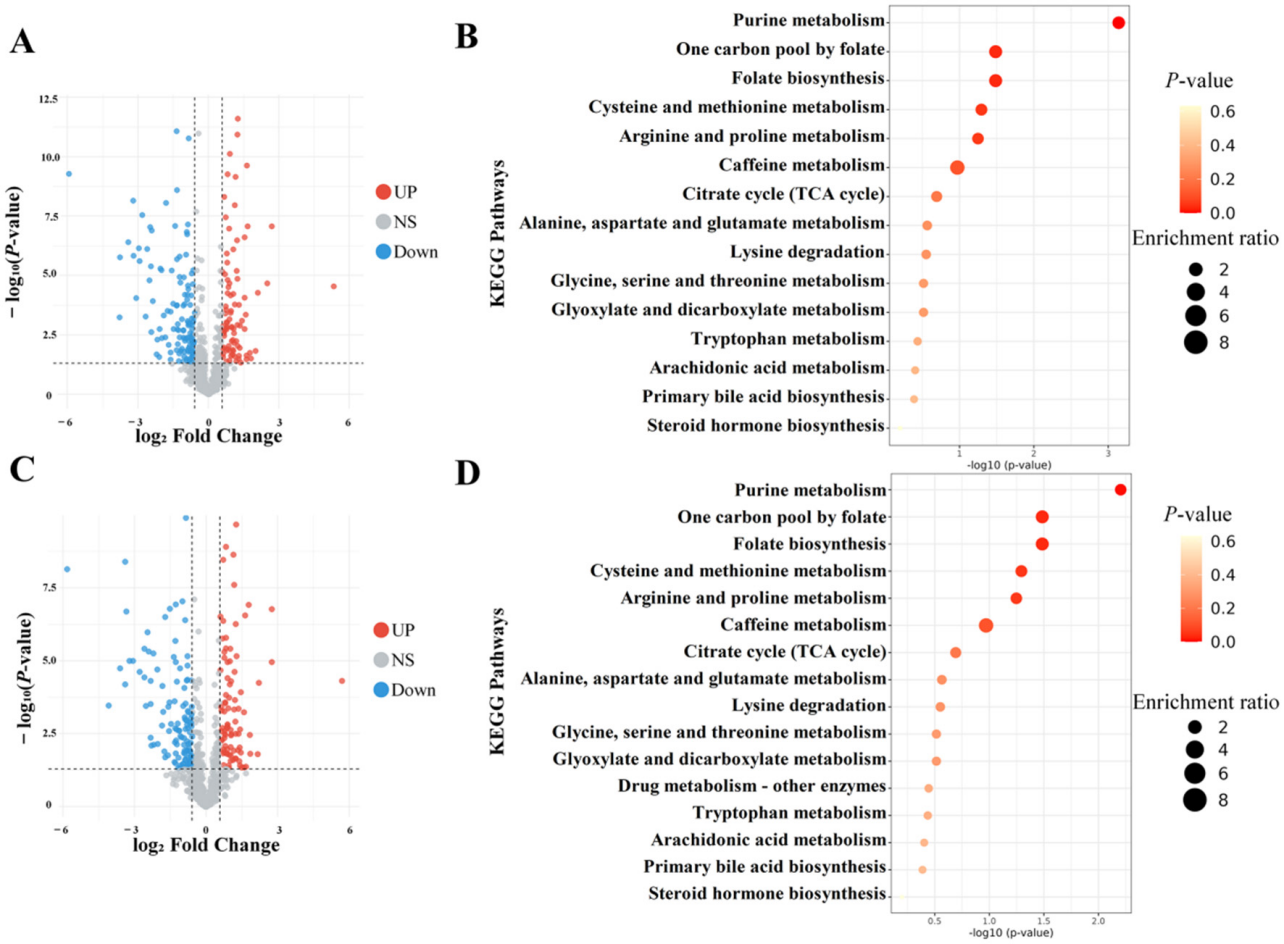
Identification of differential urinary metabolites and KEGG pathway enrichment analyses with and without covariate adjustment.**(A)** Volcano plot of differential metabolites based on unadjusted comparisons (no covariates included). **(B)** KEGG pathway enrichment bubble plot derived from the unadjusted set of differential metabolites. **(C)** Volcano plot of differential metabolites after covariate adjustment. **(D)** KEGG pathway enrichment analysis based on the covariate-adjusted differential metabolites.

To systematically identify urinary metabolites associated with CB exposure, differential metabolite screening was initially performed using stringent criteria (*P* < 0.05 and fold change > 1.5 or < 0.67), yielding 215 statistically significant differential metabolites, comprising 89 up-regulated and 126 down-regulated features (Figure 2A). Subsequent KEGG pathway enrichment analysis revealed that purine metabolism represented the most significantly enriched pathway among the identified metabolites (Figure 2B). To minimize the potential confounding effects of demographic and lifestyle factors, covariate-adjusted linear regression analyses were further conducted, controlling for age, BMI, smoking pack-years, and alcohol drinking status. This approach identified 210 differential metabolites after covariate adjustment, including 92 up-regulated and 118 down-regulated metabolites (Figure 2C), with purine metabolism consistently emerging as the top-ranked enriched pathway in the adjusted KEGG enrichment analysis (Figure 2D). The robustness of purine metabolism dysregulation across both unadjusted and covariate-adjusted analyses underscores its potential mechanistic relevance to CB exposure-induced metabolic perturbations.

Endogenous metabolites were preliminarily identified based on accurate molecular weight, characteristic fragment ions and retention time, and further annotated against the Human Metabolome Database (HMDB) and KEGG. Metabolites with reliable annotation information and significant alterations (*P* < 0.05) were selected as differential endogenous metabolites. Among the 10 significantly altered endogenous urinary metabolites associated with CB exposure (Table 4), 5 metabolites were up-regulated and 5 were down-regulated. Specifically, 3 beta-Hydroxy-5-cholestenoate (β = 2.00, *P* < 0.001), Cervonoyl ethanolamide (β = 0.90, *P* = 0.002), 11-Dehydro-thromboxane B2 (β = 0.86, *P* = 0.002), Prostaglandin E2 (PGE2, β = 0.76, *P* = 0.004), and 2-Hydroxyestrone (β = 1.13, *P* = 0.011) were up-regulated with the increase of CB exposure; Creatine (β = −0.77, *P* < 0.001), S-Adenosylhomocysteine (β = −0.75, *P* < 0.001), Citric acid (β = −0.88, *P* = 0.001), N-Acetyllactosamine (β = −0.59, *P* = 0.004), and Guanosine (β = −0.62, *P* = 0.040) were down-regulated with the increase of CB exposure.

**Table 4:** Associations between significantly altered endogenous urinary metabolites and CB exposure.

Metabolites	HMDB ID	KEGG ID	β	95%CI	*p*-value
3 beta-Hydroxy-5-cholestenoate	HMDB0012453	C17333	2.00	[1.18, 2.82]	< 0.001
Creatine	HMDB0000064	C00300	−0.77	[−1.20, −0.35]	< 0.001
S-Adenosylhomocysteine	HMDB0000939	C00021	−0.75	[−1.16, −0.33]	< 0.001
Citric acid	HMDB0000094	C00158	−0.88	[−1.39, −0.37]	0.001
Cervonoyl ethanolamide	HMDB0013627	C13828	0.90	[0.35, 1.44]	0.002
11-Dehydro-thromboxane B2	HMDB0004242	C05964	0.86	[0.32, 1.40]	0.002
N-Acetyllactosamine	HMDB0001542	C00611	−0.59	[−0.98, −0.19]	0.004
Prostaglandin E2	HMDB0001220	C00584	0.76	[0.24, 1.27]	0.004
2-Hydroxyestrone	HMDB0000343	C05298	1.13	[0.26, 2.00]	0.011
Guanosine	HMDB0000133	C00387	−0.62	[−1.22, −0.03]	0.040

Endogenous differential metabolites were annotated based on HMDB and KEGG databases, and linear regression analysis was used to explore their associations with carbon black exposure.

### 3.6 Associations between differentially expressed urinary metabolites and airway structural parameters

Airway wall area percentage of the 6th-generation airway serves as a quantitative imaging marker of airway structural remodeling, and linking altered metabolites to WA% helps elucidate the metabolic mechanism underlying CB-related small airway injury. Among the 10 differential endogenous metabolites identified in association with CB exposure, four human endogenous metabolites (Table 5) demonstrated significant correlations with the 6th-generation airway WA% (LB1+2). PGE2 exhibited a significant positive association with WA% (β = 2.78, 95% CI: 0.84–4.72, *P* = 0.006). Similarly, 3 beta-Hydroxy-5-cholestenoate was positively correlated with WA% (β = 1.39, 95% CI: 0.26–2.52, *P* = 0.017). Conversely, creatine (β = −3.48, 95% CI: −6.20–−0.76, *P* = 0.013) and citric acid (β = −2.43, 95% CI: −4.34–−0.52, *P* = 0.013) were each inversely associated with WA%. Linear regression analysis revealed a significant positive association between 3β-hydroxy-5-cholestenoate and CCAM. Although citric acid and creatine tended to be negatively associated and PGE2 tended to be positively associated with CCAM, none of these associations reached statistical significance (Table S1).

**Table 5:** Associations between differentially expressed endogenous urinary metabolites and 6th-generation airway WA%.

Metabolites	HMDB ID	β	95% CI	*p*-value
Prostaglandin E2	HMDB0001220	2.78	[0.84, 4.72]	0.006
Creatine	HMDB0000064	−3.48	[−6.20, −0.76]	0.013
Citric acid	HMDB0000094	−2.43	[−4.34, −0.52]	0.013
3 beta-Hydroxy-5-cholestenoate	HMDB0012453	1.39	[0.26, 2.52]	0.017

Associations were analyzed using linear regression adjusted for age, BMI, pack-years of smoking, and alcohol drinking status.

### 3.7 Development of a predictive model for small airway injury associated with CB exposure

**Figure 3:**
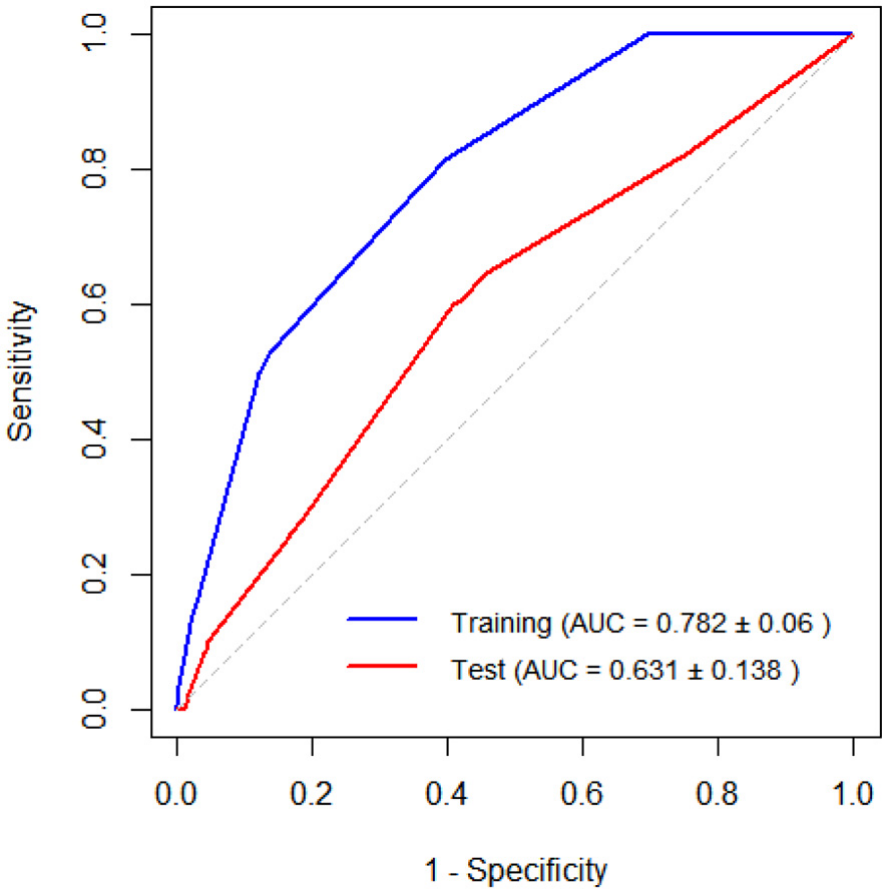
ROC curves for the KNN-based risk prediction model of small airway injury associated with CB exposure.

Small airway injury in our study was defined as a diagnostic criterion for small airway disease, with at least two of the functional parameters (MMEFP, FEF_50_P, and FEF_75_P) being less than 65% of the predicted value (22). Among the 90 participants, 13 met the criteria for small airway injury, while 77 were classified as normal. A KNN-based predictive model was developed incorporating four key urinary metabolites — PGE2, creatine, citric acid, and 3 beta-Hydroxy-5-cholestenoate — all of which were significantly associated with WA% (LB1+2), to classify CB-induced small airway injury. The dataset was randomly split into training and testing sets at a ratio of 7:3. Over 100 repeated random splits, the KNN model achieved mean AUCs of 0.782 ± 0.06 in the training set and 0.631 ± 0.138 in the test set, indicating modest predictive ability of these four metabolic biomarkers for small airway injury (Figure 3). Given the limited sample size, these findings should be considered preliminary and require external validation in larger cohorts.

## 4 Discussion

Our findings demonstrate that occupational CB exposure is significantly associated with pulmonary injury and structural deterioration of the airways, accompanied by distinct and reproducible alterations in the urinary metabolomic profile. Through untargeted urinary metabolomics analysis, we further identified a panel of core endogenous urinary metabolic biomarkers that exhibited significant correlations with airway structural parameters. By integrating these metabolic biomarkers with key confounding covariates, a robust risk prediction model for CB-related small airway injury was subsequently constructed. Collectively, these findings support the clinical feasibility of employing a noninvasive and repeatable urinary metabolomics-based approach for the early detection and surveillance of CB exposure-associated small airway impairment.

Inhalation of CB is well established as a trigger of early ventilatory dysfunction and progressive airway remodeling. Yang et al. (23) demonstrated that the average levels of FEV_1_/FVC (%), MMEF (%), FEF_25_ (%), and FEF_75_ (%) were significantly decreased in CB-exposed workers relative to unexposed controls, providing direct spirometric evidence of CB exposure-induced pulmonary functional decline. Complementing these functional observations, HRCT examination has further confirmed that airway remodeling is the predominant pathological hallmark of CB-induced small airway injury, predominantly manifested as increased airway wall thickness (11). Anatomically, the 6th -generation airway represents the proximal small airway and serves as the preferential deposition site of inhaled CB nanoparticles governed by inertial impaction, exhibiting a substantially higher deposition rate than distal 9th -generation airways; this segment therefore constitutes a critical target for the early identification and quantitative diagnosis of subclinical small airway damage via HRCT (24). Accordingly, relevant studies have verified that structural alterations in the 6th-generation airway—particularly elevated WA%—can sensitively reflect early CB-induced airway remodeling and demonstrate a significant dose-response relationship with internal CB exposure, a pattern further corroborated by the significant correlations observed in our study between WA% and urinary metabolic biomarkers. At the mechanistic level, ultrafine CB particles trigger NADPH oxidase-mediated oxidative stress, which promotes the proteolytic processing and shedding of membrane-anchored HB-EGF, thereby transactivating the EGF receptor and its downstream ERK signaling cascade. This sequence of events drives aberrant proliferation of airway epithelial cells and ultimately perpetuates the pathological processes of airway inflammation and structural remodeling (25).

Purines are fundamental biomolecules that play essential roles in genetic transmission, energy metabolism, and cell signaling, with their metabolic network encompassing *de novo* biosynthesis, salvage pathways, catabolism, and excretion to maintain physiological homeostasis (26). In the present study, urinary metabolomics analysis revealed that occupational CB exposure induced significant perturbations in purine metabolism pathway, suggesting that chronic CB inhalation may impose a substantial burden on systemic purine homeostasis. These findings are consistent with those of Chen et al. (27), who demonstrated that short-term PM_2.5_ exposure significantly perturbs the urinary purine metabolic profile, elevating levels of AMP, hypoxanthine, and succinyladenosine while reducing uric acid and adenosine phosphosulfate—a pattern indicative of accelerated purine catabolism and impaired nucleotide recycling. The pathophysiological implications of such dysregulation are considerable: disrupted purine metabolism is mechanistically linked to mitochondrial energy deficits, excessive reactive oxygen species generation, and amplified inflammatory signaling, all of which may synergistically potentiate CB-induced airway injury. Importantly, in the context of obstructive airway disease, airway levels of adenosine and AMP are significantly elevated in COPD patients and inversely correlated with FEV_1_ (%), directly implicating aberrant purine metabolism and purinergic signaling in the progression of airway pathology (28). Taken together, these lines of evidence suggest that CB exposure-driven purine metabolic dysregulation may not only serve as a sensitive early indicator of cellular stress but also actively contribute to the initiation and perpetuation of small airway structural damage through oxidative and inflammatory mechanisms.

Further urinary metabolomic analysis showed that PGE2 levels were significantly elevated in CB-exposed individuals, and its upregulation was positively correlated with increased 6th WA%, indicating a close regulatory relationship. As an abundant lipid signaling molecule and key inflammatory mediator, PGE2 is synthesized from arachidonic acid via phospholipase A2, COX and PGES, and participates in homeostasis, immune regulation and inflammation (29, 30). PGE2 is associated with COPD symptom severity and plays an important role in modulating macrophage phenotype. In particular, changes in PGE2 levels in airway samples, such as sputum, reflect local inflammatory activity in the lung, while systemic alterations further indicate its broader role in host responses (31). Furthermore, PGE2 has been shown to promote respiratory inflammation through the induction of IL-6 generation while concurrently aggravating airflow limitation in COPD via upregulation of matrix metalloproteinase expression (32, 33). Collectively, these findings suggest that CB exposure-driven PGE2 elevation may serve as a critical mediator of airway remodeling, operating through converging pathways of pro-inflammatory cytokine signaling and extracellular matrix degradation. In parallel, 3 beta-hydroxy-5-cholestenoic acid was similarly found to be significantly elevated in CB-exposed individuals, with its upregulation likewise positively correlated with increased 6th-generation WA%. As an intermediate of the cholesterol catabolic pathway, 3 beta-Hydroxy-5-cholestenoate is generated in pulmonary macrophages through CYP27A1-catalyzed hydroxylation of cholesterol to 27-hydroxycholesterol, which undergoes further oxidation to yield cholestanoic acid derivatives (34). The potential role of 3 beta-Hydroxy-5-cholestenoate in respiratory pathology remains insufficiently characterized, and dedicated mechanistic investigations are warranted to elucidate its contribution to CB exposure-associated airway injury.

Beyond lipid and sterol metabolism, perturbations in central carbon and energy metabolism were also prominently observed. Urinary citrate levels were significantly decreased in CB-exposed individuals and exhibited a significant negative correlation with 6th-generation WA%, pointing to a disruption of TCA cycle homeostasis. Disruptions in the TCA cycle are closely associated with the development of lung diseases (35). Abnormal citrate levels in COPD patients reflect a disruption of the TCA, which may be related to reduced activity of enzymes involved in oxidative energy metabolism and mitochondrial dysfunction (36). Its reduction suggests CB exposure disrupts TCA cycle homeostasis, leading to insufficient energy supply, impaired airway epithelial barrier function, and worsening injury and inflammation. Consistent with this bioenergetic deficit, creatine levels were also significantly downregulated in CB-exposed individuals and negatively correlated with 6th-generation WA%, further implicating dysregulated cellular energy buffering in the pathogenesis of small airway wall thickening. Although creatine is primarily synthesized in the liver and kidneys and serves as a phosphocreatine-based ATP regeneration reservoir in high-energy-demand tissues such as skeletal muscle, cardiac muscle, and brain, it also confers cytoprotective effects in pulmonary tissue through its antioxidant properties, independent of a primary bioenergetic role in the lung (37). CB exposure-induced creatine depletion may therefore compromise the pulmonary antioxidant defense capacity, thereby shifting the oxidative stress balance toward a pro-remodeling milieu and contributing to the progressive structural deterioration of the airways.

Based on the metabolomic results, PGE2, citric acid, creatine and 3 beta-Hydroxy-5-cholestenoate were screened as the key differential metabolites. Using these four metabolites as predictors, we built a KNN-based risk model for early small airway injury linked to CB exposure. KNN offers a simple, non-parametric, and intuitively appealing alternative to more complex machine learning algorithms (38), delivering comparable predictive performance in certain contexts without the added complexity of hyperparameter tuning or explicit model training. The present KNN model demonstrated modest discriminative performance, with a test set AUC of 0.631 ± 0.138, suggesting that these four urinary metabolites may hold preliminary value as candidate biomarkers for CB-related small airway injury. While these findings are encouraging, they should be interpreted with caution given the limited sample size, and the model's clinical utility remains to be confirmed through external validation in larger independent cohorts.

Several limitations of the present study warrant acknowledgment. First, the relatively modest sample size constrains the statistical power of the predictive model and may limit its generalizability; external validation in larger, more ethnically and occupationally diverse CB-exposed cohorts is therefore necessary before the model can be translated into clinical or occupational health practice. Second, the cross-sectional study design precludes the establishment of causal or temporal relationships between the identified core metabolites and small airway structural injury, and the directionality of these associations remains to be determined. Finally, the relatively low number of positive cases relative to the total sample size raises concerns regarding model instability and potential overfitting in the KNN classifier, as reflected by the discrepancy between training and test set AUC values, and underscores the need for prospective replication in larger cohorts. To address these limitations, future studies should enroll larger sample sizes spanning a wider spectrum of CB exposure levels, incorporate multi-omics data integration to elucidate underlying biological mechanisms, and conduct longitudinal follow-up to establish temporality and strengthen causal inference, ultimately contributing to more robust occupational health surveillance frameworks for particulate matter-exposed workers.

## Data Availability

The data that support the findings of this study are available from the corresponding author upon reasonable request.

## References

[1] Chaudhuri I Fruijtier-Pölloth C Ngiewih Y Levy L. Evaluating the evidence on genotoxicity and reproductive toxicity of carbon black: a critical review. Crit Rev Toxicol. (2018) 48:143–69. doi: 10.1080/10408444.2017.1391746 29095661

[2] Zainol MM Zainal Abidin A Mustaffa N Syed Abdul Rahman SNF Mohd Azli AA Mustapa AN . A review on carbon black production, properties, and its applications toward energy storage. Energy. (2025) 340:139123. doi: 10.1016/j.energy.2025.139123

[3] Robertson CG Hardman NJ. Nature of carbon black reinforcement of rubber: perspective on the original polymer nanocomposite. Polymers. (2021) 13:538. doi: 10.3390/polym13040538 33673094 PMC7917815

[4] Salahuddin B Faisal SN Baigh TA Alghamdi MN Islam MS Song B . Carbonaceous materials coated carbon fibre reinforced polymer matrix composites. Polymers. (2021) 13:2771. doi: 10.3390/polym13162771 34451310 PMC8399309

[5] Wilbourn J Partensky C Morgan WG. IARC evaluates printing processes and printing inks, carbon black and some nitro compounds. Scand J Work Environ Health. (1996) 22:154–6. 8738896

[6] Tang J Cheng W Gao J Li Y Yao R Rothman N . Occupational exposure to carbon black nanoparticles increases inflammatory vascular disease risk: an implication of an ex vivo biosensor assay. Part Fibre Toxicol. (2020) 17:47. doi: 10.1186/s12989-020-00378-8 32993720 PMC7523398

[7] Szozda R. Pneumoconiosis in carbon black workers. J uoeh. (1996) 18:223–8. doi: 10.7888/juoeh.18.223 8829263

[8] Saputra D Yoon JH Park H Heo Y Yang H Lee EJ . Inhalation of carbon black nanoparticles aggravates pulmonary inflammation in mice. Toxicol Res. (2014) 30:83–90. doi: 10.5487/TR.2014.30.2.083 25071917 PMC4112069

[9] Bao L Liu Q Wang J Shi L Pang Y Niu Y . The interactions of subcellular organelles in pulmonary fibrosis induced by carbon black nanoparticles: a comprehensive review. Arch Toxicol. (2024) 98:1629–43. doi: 10.1007/s00204-024-03719-0 38536500

[10] Gardiner K van Tongeren M Harrington M. Respiratory health effects from exposure to carbon black: results of the phase 2 and 3 cross sectional studies in the European carbon black manufacturing industry. Occup Environ Med. (2001) 58:496–503. doi: 10.1136/oem.58.8.496 11452043 PMC1740179

[11] Cao X Lin L Sood A Ma Q Zhang X Liu Y . Small airway wall thickening assessed by computerized tomography is associated with low lung function in chinese carbon black packers. Toxicological sciences. (2020) 178:26–35. doi: 10.1093/toxsci/kfaa134 32818265 PMC7825005

[12] Toumpanakis D Kim Y Usmani OS. Small airways disease in patients with COPD: a question-and-answer approach for everyday clinical practice. Chest. (2026) 169:641–51. doi: 10.1016/j.chest.2025.07.002 40675548 PMC12975394

[13] Graham BL Steenbruggen I Miller MR Barjaktarevic IZ Cooper BG Hall GL . Standardization of spirometry 2019 update. An official American thoracic society and european respiratory society technical statement Am J Respir Crit Care Med. (2019) 200:e70–88. doi: 10.1164/rccm.201908-1590ST 31613151 PMC6794117

[14] Purohit S Dutt N Saini L Panwar R Kumar S. High resolution computed tomography in chronic obstructive pulmonary disease patients: do not forget radiation hazard. Lung India. (2016) 33:582–3. doi: 10.4103/0970-2113.189006 27625468 PMC5006354

[15] Qiu S Cai Y Yao H Lin C Xie Y Tang S . Small molecule metabolites: discovery of biomarkers and therapeutic targets. Signal Transduct Target Ther. (2023) 8:132. doi: 10.1038/s41392-023-01399-3 36941259 PMC10026263

[16] Wang L Tang Y Liu S Mao S Ling Y Liu D . Metabonomic profiling of serum and urine by H NMR-based spectroscopy discriminates patients with chronic obstructive pulmonary disease and healthy individuals. PLoS One. (2013) 8:e65675. doi: 10.1371/journal.pone.0065675 23755267 PMC3675021

[17] Papamichael MM Katsardis C Sarandi E Georgaki S Frima ES Varvarigou A . Application of metabolomics in pediatric asthma: prediction, diagnosis and personalized treatment. Metabolites. (2021) 11:251. doi: 10.3390/metabo11040251 33919626 PMC8072856

[18] Liang D Li Z Vlaanderen J Tang Z Jones DP Vermeulen R . A state-of-the-science review on high-resolution metabolomics application in air pollution health research: current progress, analytical challenges, and recommendations for future direction. Environ Health Perspect. (2023) 131:56002. doi: 10.1289/EHP11851 37192319 PMC10187974

[19] Wang T Li J Liang Y Han W Tang J Cheng G . Joint effects of carbon black exposure and dietary antioxidant vitamin intake on small airway dysfunction. Front Nutr. (2021) 8:716398. doi: 10.3389/fnut.2021.716398 34760908 PMC8572798

[20] Zhang R Dai Y Zhang X Niu Y Meng T Li Y . Reduced pulmonary function and increased pro-inflammatory cytokines in nanoscale carbon black-exposed workers. Part Fibre Toxicol. (2014) 11:73. doi: 10.1186/s12989-014-0073-1 25497989 PMC4318129

[21] Cheng W Liu Y Tang J Duan H Wei X Zhang X . Carbon content in airway macrophages and genomic instability in Chinese carbon black packers. Arch Toxicol. (2020) 94:761–71. doi: 10.1007/s00204-020-02678-6 32076763

[22] Xu L Sgalla G Wang F Zhu M Li L Li P . Monitoring small airway dysfunction in connective tissue disease-related interstitial lung disease: a retrospective and prospective study. BMC Pulm Med. (2023) 23:90. doi: 10.1186/s12890-023-02381-z 36941622 PMC10026226

[23] Yang M Li Y Meng T Zhang L Niu Y Dai Y . Ultrafine CB-induced small airway obstruction in CB-exposed workers and mice. Sci Total Environ. (2019) 671:866–73. doi: 10.1016/j.scitotenv.2019.03.033 30947057

[24] Cheng W Ma Z Zhang X Zheng Y Han W Yu Y . Deposition patterns of carbon black nanoparticles in pulmonary lobes and airway generations: implications for lung remodeling. Environ Sci Technol. (2026) 60:13848–58. doi: 10.1021/acs.est.5c14667 41945664

[25] Tamaoki J Isono K Takeyama K Tagaya E Nakata J Nagai A. Ultrafine carbon black particles stimulate proliferation of human airway epithelium via EGF receptor-mediated signaling pathway. Am J Physiol Lung Cell Mol Physiol. (2004) 287:L1127–33. doi: 10.1152/ajplung.00241.2004 15298855

[26] Zhao H French JB Fang Y Benkovic SJ. The purinosome, a multi-protein complex involved in the de novo biosynthesis of purines in humans. Chem Commun (Camb). (2013) 49:4444–52. doi: 10.1039/c3cc41437j 23575936 PMC3877848

[27] Chen C Li H Niu Y Liu C Lin Z Cai J . Impact of short-term exposure to fine particulate matter air pollution on urinary metabolome: a randomized, double-blind, crossover trial. Environ Int. (2019) 130:104878. doi: 10.1016/j.envint.2019.05.072 31200160

[28] Esther CR. Jr., Lazaar AL, Bordonali E, Qaqish B, Boucher RC. Elevated airway purines in COPD. Chest. (2011) 140:954–60. doi: 10.1378/chest.10-2471 21454402 PMC3186686

[29] Bozyk PD Moore BB. Prostaglandin E2 and the pathogenesis of pulmonary fibrosis. Am J Respir Cell Mol Biol. (2011) 45:445–52. doi: 10.1165/rcmb.2011-0025RT 21421906 PMC3175580

[30] Wang B Wu L Chen J Dong L Chen C Wen Z . Metabolism pathways of arachidonic acids: mechanisms and potential therapeutic targets. Signal Transduct Target Ther. (2021) 6:94. doi: 10.1038/s41392-020-00443-w 33637672 PMC7910446

[31] Tejwani V Villabona-Rueda AF Khare P Zhang C Le A Putcha N . Airway and systemic prostaglandin E2 association with COPD symptoms and macrophage phenotype. Chronic Obstr Pulm Dis. (2023) 10:159–69. doi: 10.15326/jcopdf.2022.0375 36976551 PMC10392871

[32] Lin CC Lee IT Yang YL Lee CW Kou YR Yang CM. Induction of COX-2/PGE/IL-6 is crucial for cigarette smoke extract-induced airway inflammation: role of TLR4-dependent NADPH oxidase activation. Free Radic Biol Med. (2010) 48:240–54. doi: 10.1016/j.freeradbiomed.2009.10.047 19892012

[33] Chen Y Chen P Hanaoka M Droma Y Kubo K. Enhanced levels of prostaglandin E2 and matrix metalloproteinase-2 correlate with the severity of airflow limitation in stable COPD. Respirology. (2008) 13:1014–21. doi: 10.1111/j.1440-1843.2008.01365.x 18699805

[34] Meaney S Bonfield TL Hansson M Babiker A Kavuru MS Thomassen MJ. Serum cholestenoic acid as a potential marker of pulmonary cholesterol homeostasis: increased levels in patients with pulmonary alveolar proteinosis. J Lipid Res. (2004) 45:2354–60. doi: 10.1194/jlr.M400302-JLR200 15466366

[35] Lu X Zhang A Wang H Xu X Chen L Luo L. Emerging role of the TCA cycle and its metabolites in lung disease. Front Physiol. (2025) 16:1621013. doi: 10.3389/fphys.2025.1621013 40895429 PMC12394498

[36] Ran N Pang Z Gu Y Pan H Zuo X Guan X . An updated overview of metabolomic profile changes in chronic obstructive pulmonary disease. Metabolites. (2019) 9:111 doi: 10.3390/metabo9060111 31185592 PMC6631716

[37] De Benedetto F Pastorelli R Ferrario M de Blasio F Marinari S Brunelli L . Supplementation with Qter(^®^) and creatine improves functional performance in COPD patients on long term oxygen therapy. Respir Med. (2018) 142:86–93. doi: 10.1016/j.rmed.2018.08.002 30170808

[38] Prasath ST Navaneethan C. Colorectal cancer prognosis based on dietary pattern using synthetic minority oversampling technique with K-nearest neighbors approach. Sci Rep. (2024) 14:17709. doi: 10.1038/s41598-024-67848-3 39085324 PMC11292025

