# Supplementary material for: Urinary metabolomics reveals potential biomarkers for monitoring carbon black exposure-related airway injury
Source: Front Public Health. 2026 Jun 25;14:1809269. doi: 10.3389/fpubh.2026.1809269 (PMC13346074; doi:10.3389/fpubh.2026.1809269)
Supplement: Supplementary file 1 [file Data_Sheet_1.docx]

**Urinary Metabolomics Reveals Potential Biomarkers for Monitoring Carbon Black Exposure-Related Airway Injury**

Rou Wen†^1, 2^, Zijing Wu†^1, 2^, Zhaohui Mu†^3^, Jianzhong Zhang^2^, Yaozu Han^2^, Yixuan Wang^4^, Jinglong Tang^5^, Yuxin Zheng^5^*, Wei Han^2, 3^*, Weiwei Qin^6^*

^1^School of Medicine and Pharmacy, Ocean University of China

^2^Qingdao Key Laboratory of Respiratory Comorbidity Remodeling and Precision Prevention, Qingdao Hospital, University of Health and Rehabilitation Sciences (Qingdao Municipal Hospital), Qingdao 266071, China

^3^Department of Respiratory and Critical Care Medicine, Qingdao Hospital, University of Health and Rehabilitation Sciences (Qingdao Municipal Hospital), Qingdao 266071, China

^4^Central Laboratory, Qingdao Hospital, University of Health and Rehabilitation Sciences (Qingdao Municipal Hospital), Qingdao 266071, China

^5^Department of Environmental and Occupational Health, School of Public Health, Qingdao University, Qingdao 266071, China

^6^Department of Anesthesiology, Qingdao Hospital, University of Health and Rehabilitation Sciences (Qingdao Municipal Hospital), Qingdao, 266071, China

†These authors have contributed equally to this work and share first authorship.

* Correspondence: Weiwei Qin, weiweiqin@163.com; Wei Han, sallyhan1@163.com; Yuxin Zheng, yxzheng@qdu.edu.cn

**
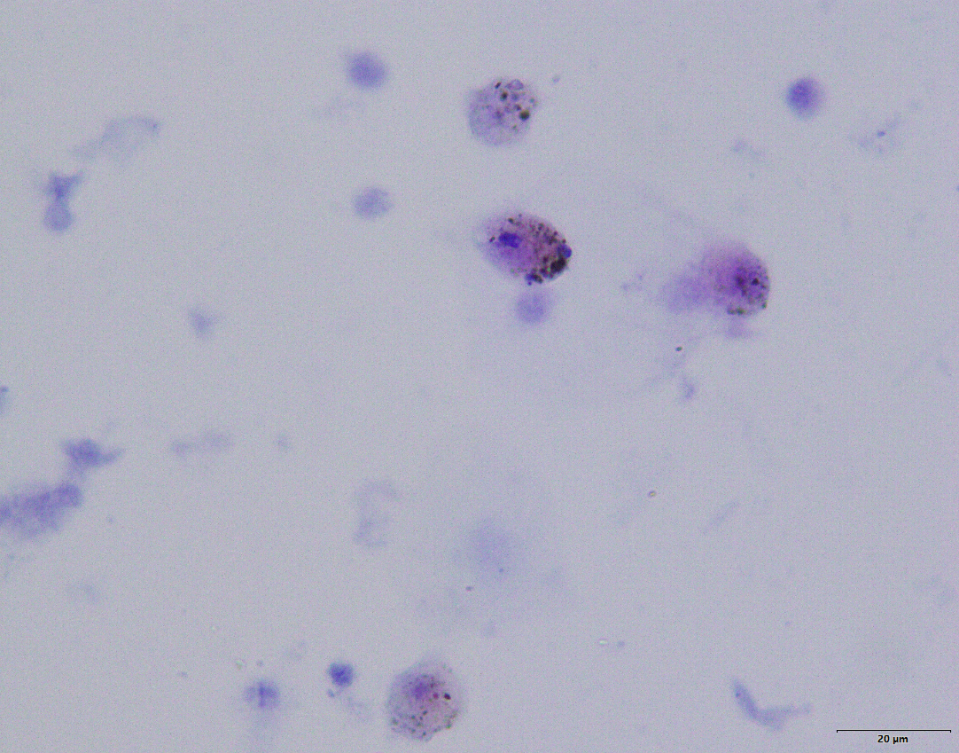
**

**Figure S1. Representative image of a macrophage with CB deposition.**

**Table S1. Associations of core endogenous urinary metabolites with CCAM.**

| Metabolite Name | HMDB ID | β | 95% CI | *P*-value |
| --- | --- | --- | --- | --- |
| Prostaglandin E2 | HMDB0001220 | 0.027 | [-0.004, 0.058] | 0.086 |
| Citric acid | HMDB0000094 | -0.032 | [-0.070, 0.005] | 0.089 |
| Creatine | HMDB0000064 | -0.026 | [-0.058, 0.005] | 0.101 |
| 3 beta-Hydroxy-5-cholestenoate | HMDB0012453 | 0.104 | [0.040, 0.169] | 0.002 |

Note: Associations were analyzed using linear regression adjusted for age, BMI, pack-years of smoking, and alcohol drinking status.
